# Dietary and Pharmacological Modulation of Aging-Related Metabolic Pathways: Molecular Insights, Clinical Evidence, and a Translational Model

**DOI:** 10.3390/ijms26199643

**Published:** 2025-10-02

**Authors:** Antonio Fernando Murillo-Cancho, David Lozano-Paniagua, Bruno José Nievas-Soriano

**Affiliations:** 1 Department of Nursing, Physiotherapy and Medicine, Faculty of Health Sciences, University of Almeria, 04120 Almeria, Spain; brunonievas@ual.es; 2 Faculty of Health Sciences, International University of La Rioja, 26006 Logroño, Spain

**Keywords:** caloric restriction, intermittent fasting, caloric restriction mimetics, mTOR, AMPK, sirtuins, autophagy, precision nutrition, healthy longevity

## Abstract

Advances in geroscience suggest that aging is modulated by molecular pathways that are amenable to dietary and pharmacological intervention. We conducted an integrative critical review of caloric restriction (CR), intermittent fasting (IF), and caloric restriction mimetics (CR-mimetics) to compare shared mechanisms, clinical evidence, limitations, and translational potential. Across modalities, CR and IF consistently activate AMP-activated protein kinase and sirtuins, inhibit mTOR (mechanistic target of rapamycin) signaling, and enhance autophagy, aligning with improvements in insulin sensitivity, lipid profile, low-grade inflammation, and selected epigenetic aging measures in humans. CR-mimetics, such as metformin, resveratrol, rapamycin, and spermidine, partially reproduce these effects; however, long-term safety and efficacy in healthy populations remain incompletely defined. Methodological constraints—short trial duration, selective samples, intermediate (nonclinical) endpoints, and limited adherence monitoring—impede definitive conclusions on hard outcomes (frailty, disability, hospitalization, mortality). We propose the Active Management of Aging and Longevity (AMAL) model, a three-level biomarker-guided framework that integrates personalized diet, chrono-nutrition, exercise, and the selective use of CR-mimetics, along with digital monitoring and decision support. AMAL emphasizes epigenetic clocks, multi-omics profiling, inflammatory and microbiome metrics, and adaptive protocols to enhance adherence and clinical relevance. Overall, CR, IF, and CR mimetics constitute promising, complementary strategies to modulate biological aging; rigorous long-term trials with standardized biomarkers and clinically meaningful endpoints are needed to enable their scalable implementation.

## 1. Introduction

The increase in life span over recent decades has led to an unprecedented demographic phenomenon: the progressive aging of the global population. This transformation, driven by reduced mortality and advances in healthcare, entails a significant rise in chronic diseases, disability, and functional dependence, thereby placing an increasing burden on healthcare systems and creating an urgent need for effective preventive strategies across the life course [1,2]. In response to this challenge, international agencies such as the WHO and the United Nations have developed action frameworks to promote healthy aging, with an emphasis on preserving functional capacity and autonomy in older age [3].

From a physiological perspective, aging has traditionally been understood as an inevitable and irreversible process. However, biomedical research over the past two decades has revealed that this process is regulated by identifiable and, to some extent, modifiable molecular mechanisms [4]. This paradigm shift has given rise to the concept of geroscience, which connects biological aging with the onset and progression of chronic diseases, and posits that targeting aging mechanisms could simultaneously delay multiple age-related pathologies [4].

Within this framework, a robust explanatory model has been formulated that describes the molecular pillars of aging, known as the “hallmarks of aging” [5]. These include epigenetic alterations, mitochondrial dysfunction, genomic instability, loss of proteostasis, cellular senescence, stem cell exhaustion, immunosenescence, and, more recently, gut dysbiosis as a key systemic integrator [6,7]. A detailed understanding of these mechanisms has enabled the identification of therapeutic targets with the potential to slow down aging and extend the health span. Moreover, this knowledge has facilitated the identification of novel biomarkers of biological aging, including epigenetic clocks, which quantify the rate of individual aging and enable the evaluation of anti-aging interventions [8].

Diet thus emerges as a strategic modulator of healthy aging. Longevity-related metabolic pathways—such as AMPK, mTOR, sirtuins, or FOXO—respond directly to dietary, energetic, and chronobiological stimuli, and are involved in processes such as autophagy, mitochondrial function, proteostasis, and antioxidant responses [9,10]. This knowledge has fostered the development of strategies specifically designed to influence cellular and systemic aging. Among these, energy restriction strategies have gained increasing interest as tools to promote longevity and functional health, particularly two approaches: caloric restriction (CR) and intermittent fasting (IF), both of which have documented effects in animal models and, to a lesser extent, in human clinical studies [11,12].

CR, defined as a controlled reduction in caloric intake without malnutrition, has been shown to significantly extend lifespan across multiple species, from yeast to non-human primates [11]. In humans, studies such as CALERIE (Comprehensive Assessment of Long-term Effects of Reducing Intake of Energy) have demonstrated improvements in cardiometabolic markers and reductions in epigenetic aging rates [8]. Nevertheless, CR presents substantial limitations in terms of clinical feasibility, long-term adherence, and potential risks, such as sarcopenia or bone mass loss, in vulnerable populations [13].

By contrast, IF encompasses a range of protocols that alternate periods of feeding and fasting, without necessarily reducing total energy intake [14]. This strategy has demonstrated benefits in insulin sensitivity, lipid profile, blood pressure, and low-grade inflammation [15], with the additional advantage of aligning with circadian rhythms, which may enhance its physiological efficacy [16]. However, its clinical applicability also faces significant challenges, including methodological heterogeneity, variability in individual responses, and the scarcity of studies with robust clinical outcomes [17].

In parallel, other approaches have gained relevance through the use of so-called CR mimetics, bioactive compounds capable of inducing effects similar to energy restriction by activating common molecular pathways, without the need for strict dietary modification [18,19]. Some of the most studied include metformin, resveratrol, berberine, and rapamycin, which are emerging as complementary therapeutic options, especially in individuals with poor adherence or contraindications to strict dietary interventions [20].

Building on the shared mechanisms and intermediate clinical outcomes observed with CR, IF, and selected mimetics, we propose a conceptual framework (AMAL) that organizes these strategies into levels of personalization. This model is presented as a working hypothesis for future clinical validation and does not imply that comprehensive lifestyle interventions have been evaluated in the studies analyzed in this context. This review examines the molecular and clinical foundations of CR, IF, and CR mimetics, providing the basis for this model.

Heterogeneous study designs, moderate durations, and a predominant reliance on intermediate outcomes characterize the available evidence. Therefore, this review adopts a comparative and integrative approach to position CR, IF, and CR mimetics within their translational context, without overinterpreting their findings.

Moreover, most existing reviews adopt a purely descriptive or narrative approach, without critically integrating evidence from a translational perspective that facilitates real-world clinical implementation. This gap is particularly relevant given the urgent need to design personalized dietary strategies tailored to the physiological, metabolic, and functional profiles of different age groups or risk categories [8,9,13,20,21]. New proposals must incorporate both phenotypic criteria and molecular biomarkers of aging, insulin resistance, or oxidative stress, enabling individualized interventions and improving efficacy in populations with cardiometabolic risk [22].

The link between accelerated aging and metabolic diseases such as type 2 diabetes, sarcopenic obesity, or NAFLD (non-alcoholic fatty liver disease) has become increasingly evident. Recent studies demonstrate that modulation of pathways such as the IGF-1/mTOR axis or regulation of the IL-6/NF-κB inflammatory axis through dietary intervention may exert positive effects not only on aging biomarkers but also on clinical outcomes in these diseases [23,24,25]. In this regard, the integration of nutritional intervention into precision medicine models is clinically useful only when it incorporates systemic metabolic data from the patient (e.g., glucose, lipids, insulin resistance, low-grade inflammation) and, when available, aging-related biomarkers. With this information, artificial intelligence tools can support patient stratification and response prediction, always as a complement to clinical evidence, not as a substitute for it [26,27].

Accordingly, this manuscript provides an integrative critical review of the available evidence on CR, IF, and mimetic strategies to identify their shared molecular mechanisms, assess their real-world clinical applicability, and propose a personalized, multidisciplinary, and clinically viable intervention model. Particular attention is given to the role of molecular biomarkers with predictive or therapeutic value. Relevant serum markers include fasting glucose, HbA1c, HOMA-IR (homeostatic model assessment of insulin resistance), TG/HDL-C ratio, apoB, hs-CRP, and IL-6, as well as adipokines and liver enzymes, which are widely used for metabolic risk stratification. Aging-related biomarkers, such as DNA methylation clocks and circulating GDF-15, provide complementary information, typically showing higher levels of IL-6/CRP and GDF-15, and lower levels of IGF-1, in older adults compared with younger populations. Across interventions, CR, IF, and CR mimetics share improvements in insulin sensitivity, lipid profile, and low-grade inflammation. More specific effects include lower IGF-1 with CR, higher ketones with IF, and anti-inflammatory or autophagy-related signals with selected mimetics [11,12,22].

This review synthesizes mechanistic insights and intermediate human outcomes, avoiding inferences regarding mortality, disability, or major clinical events, which require longer-term trials.

## 2. Materials and Methods

### 2.1. Study Design

We conducted an integrative critical review of scientific literature, focusing on three dietary strategies associated with modulating human aging: caloric restriction (CR), intermittent fasting (IF), and caloric restriction mimetics. The objective was to identify similarities and differences in their molecular mechanisms, clinical evidence, limitations of applicability, and potential synergies from a translational and personalized perspective.

This approach was chosen for its ability to combine different types of evidence (preclinical, clinical, prior reviews), allowing for a more contextualized analysis than that provided by traditional systematic reviews, particularly in a field characterized by high methodological heterogeneity and limited availability of robust clinical trials.

### 2.2. Search Strategy

A comprehensive search was conducted in the PubMed, Scopus, and Web of Science databases, spanning the period from 1 January 2004 to 31 March 2025 (last search: 31 March 2025). We combined MeSH descriptors and free-text terms related to caloric restriction (“caloric restriction,” “dietary restriction”), intermittent fasting (“intermittent fasting,” “time-restricted feeding,” “alternate-day fasting”), and caloric restriction mimetics (metformin, resveratrol, rapamycin, spermidine, berberine), together with descriptors for aging and longevity (“aging,” “longevity,” “metabolic health”).

An example of a PubMed query was: (“caloric restriction” OR “dietary restriction” OR “energy restriction”) OR (“intermittent fasting” OR “time-restricted feeding” OR “alternate-day fasting”) OR (“caloric restriction mimetics” OR metformin OR resveratrol OR rapamycin OR spermidine OR berberine) AND (“aging” OR “longevity” OR “metabolic health”). Search strategies were adapted for Scopus and WoS, utilizing equivalent descriptors. Filters were applied for clinical trials, systematic reviews, human studies, and full-text articles in English or Spanish. Full, reproducible search details by database are provided in Supplementary Materials Table S1 (search).

### 2.3. Inclusion and Exclusion Criteria

Studies were included if they met the following criteria:

- Clinical studies in humans and systematic reviews with a comparative focus on caloric restriction (CR), intermittent fasting (IF), or CR mimetics (e.g., metformin, resveratrol, rapamycin, spermidine, FMD) with defined protocols.
- Preclinical studies only if they provide a straightforward pathophysiological extrapolation to humans.
- Reporting of molecular mechanisms (mTOR, AMPK, SIRT, IGF-1, autophagy) and/or biomarkers (epigenetic, transcriptomic, metabolomic, or clinically relevant).

Excluded were:

- Animal studies without clinical or pathophysiological extrapolation to humans.
- Opinion papers, editorials, letters to the editor, and conference abstracts.
- Reviews lacking a critical component or comparative analysis between CR, IF, and mimetics.
- Articles focused exclusively on other diets (e.g., ketogenic, DASH, Mediterranean) without molecular links to longevity mechanisms.

We included original studies (clinical trials, cohorts, and relevant preclinical research) and critical reviews addressing the relationship between CR, IF, or mimetics and aging, longevity, or metabolic health. Opinion papers lacking empirical support, dietary interventions without aging outcomes, duplicates, and animal studies without translational relevance were excluded. Examples of exclusions included narrative reviews without updated references, short-term rodent studies without aging biomarkers, and trials focused solely on weight loss without aging parameters.

### 2.4. Selection Process

**Figure 1:**
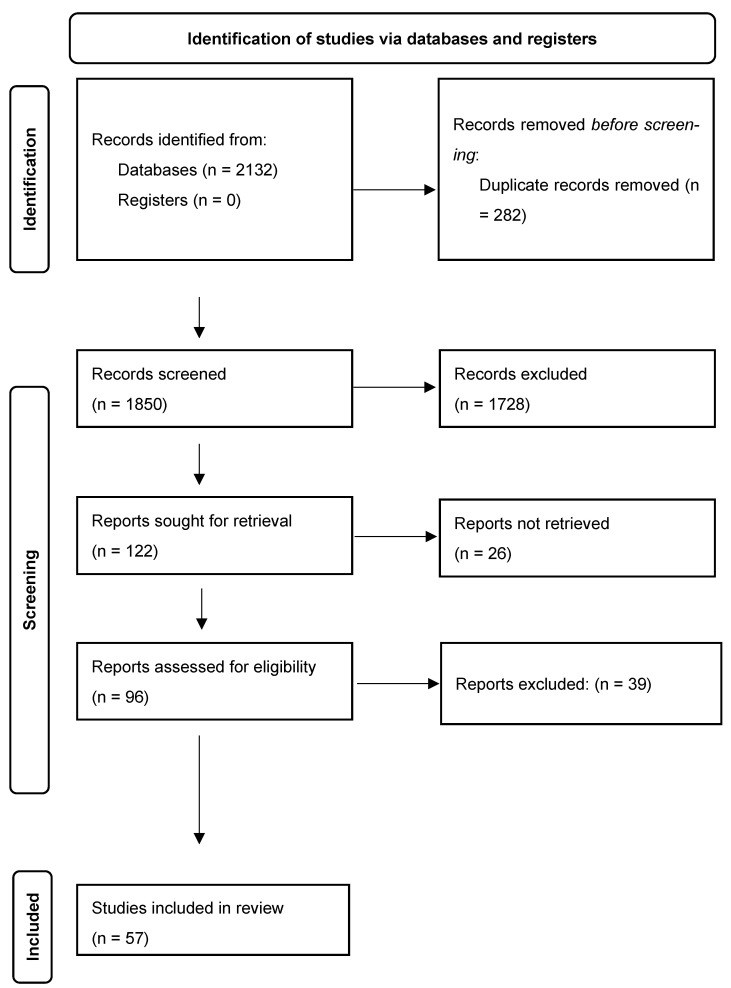
PRISMA flow diagram of the study selection process.

After duplicate removal, two reviewers independently screened titles and abstracts; potentially eligible records were assessed in full text. Discrepancies were resolved by consensus, and when disagreement persisted, a third reviewer acted as arbiter. The identification, screening, and selection process is presented in Figure 1, following PRISMA flowchart guidelines.

From a total of 2132 initial records, 282 duplicates were removed, leaving 1850 titles/abstracts for screening, of which 1728 were excluded. Ninety-six articles were assessed in full text, with 39 exclusions, resulting in 57 studies included in the qualitative synthesis. Relevance was determined by using a structured comparative approach based on:

- Level of evidence and type of population studied.
- Methodological quality, replicability, and control of confounding variables.
- Clinical relevance of outcomes and biomarkers.
- Consistency between described molecular mechanisms and therapeutic applicability.
- Suitability for current clinical contexts and potential for intervention personalization.

The findings were thematically organized according to the type of intervention (CR, IF, mimetics) and structured through comparative tables synthesizing study type, design, primary outcomes, and translational applicability.

The search strategy and selection process enabled the identification of studies that constitute the analytical corpus of this integrative critical review. These works include controlled clinical trials, observational studies with translational application, systematic reviews with a comparative focus, and preclinical studies with high pathophysiological relevance to humans. Complete information for the 57 included studies is provided in Supplementary Material Table S1.

Priority was given to the inclusion of investigations with clearly defined interventions in caloric restriction (CR), intermittent fasting (IF), and CR mimetics, as well as those incorporating molecular biomarkers of aging or clinical–functional parameters with implementation potential. Within the analyzed body of literature, structured clinical trials such as the CALERIE studies [17,23] stand out, documenting the effects of moderate CR on cardiometabolic health, epigenetic aging, and immunometabolic function in humans, with their methodological quality establishing them as key references in the field of nutritional geroscience.

Recent systematic reviews providing comparative syntheses of IF effects on glycemic, lipid, and body composition parameters were also included [24,25]. In parallel, studies focused on CR mimetics that evaluated immunological, functional, or epigenetic outcomes were considered [26,27].

Additionally, studies examining the utility of epigenetic (DunedinPACE, GrimAge), transcriptomic, or metabolomic biomarkers in assessing aging and responses to dietary interventions were included [28,29]. This methodologically diverse selection enables a critical and contextualized comparison of the major dietary strategies, their shared molecular mechanisms, and their actual clinical implications.

All studies were systematically categorized according to the type of intervention (CR, IF, mimetics), methodological design (clinical trial, systematic review, observational study, preclinical study with human extrapolation), and principal findings. This classification is summarized in Table 1 and serves as the foundation for the comparative analysis presented in the following sections of the manuscript.

### 2.5. Methodological Limitations

As this is a critical narrative review, no meta-analysis or quantitative bias assessment was performed. The following limitations are acknowledged:

- High heterogeneity among study designs, populations, and outcomes.
- Limited availability of longitudinal studies with robust clinical biomarkers.
- Lack of standardization in the definition of interventions (CR, IF, mimetics).
- Possible overlap of interventions or outcomes among studies.

Nevertheless, these limitations are inherent to an emerging field of research and justify the need for a critical and integrative approach such as the one proposed here. Despite these challenges, efforts were made to ensure thematic representativeness and comparative rigor across the different interventions. This approach enables a reflective and applied synthesis, which helps develop personalized clinical strategies in healthy aging.

Epigenetic biomarkers are considered here as exploratory indicators of aging pace; their use as primary outcomes requires further standardization and replication.

## 3. Synthesis of the Evidence

The literature search and screening process identified a total of 57 studies that met the inclusion criteria, with detailed information provided in Supplementary Material Table S1. To facilitate interpretation and highlight the most relevant findings, summary tables are presented in the main text, grouping the most significant studies included in the integrative critical review by intervention, design, and primary outcomes (Table 1).

In addition, the evidence for the different interventions is organized into three blocks: caloric restriction (Table 2), intermittent fasting (Table 3), and caloric restriction mimetics (Table 4), along with a final comparative table (Table 5). These tables summarize the most representative clinical and preclinical studies, their key methodological features, and critical results regarding biomarkers of aging, metabolic health, and functional outcomes. This organization provides a comparative overview of the different strategies, which will subsequently be integrated and critically discussed within the framework of the proposed AMAL model.

This table summarizes representative studies on caloric restriction (CR), intermittent fasting (IF), and CR mimetics (CRM), including conceptual reviews, clinical and preclinical research, and large integrative frameworks. Each study is characterized by its primary topic, design type, and the most significant findings related to aging, longevity, or metabolic health.

### 3.1. Caloric Restriction (CR)

Caloric restriction (CR), defined as a sustained reduction in energy intake without inducing malnutrition, has been extensively studied in animal models for its capacity to extend lifespan and reduce the incidence of age-related chronic diseases [13,53]. These adaptations include improvements in insulin sensitivity, reductions in oxidative stress, activation of autophagy, and modulation of molecular pathways such as AMPK, mTOR, and sirtuins [13,18,30].

In humans, the CALERIE program (Comprehensive Assessment of Long-term Effects of Reducing Intake of Energy) constitutes the primary source of clinical evidence. In its first phase, Weiss et al. demonstrated that a 25% CR maintained for six months significantly improved glucose tolerance, enhanced insulin sensitivity, and reduced C-reactive protein (CRP) levels in healthy adults [15]. Subsequently, Most et al. confirmed that a two-year intervention achieved sustained reductions in blood pressure, LDL (low-density lipoprotein) cholesterol, TNF-α, and plasma insulin, even in non-obese individuals [16].

In addition, Martin et al. evaluated the functional effects of CR in healthy adults, reporting improvements in mood, sleep quality, and sexual function [17], whereas Racette et al. characterized long-term adherence and retention in study participants, showing good clinical feasibility under controlled conditions [54].

A key advance has been the incorporation of molecular biomarkers into the assessment of aging. Waziry et al. reported a significant deceleration in the rate of biological aging, as measured by epigenetic clocks such as DunedinPACE, after 24 months of CR [23]. These findings are complemented by transcriptomic studies showing favorable regulation of genes involved in cellular resilience and metabolism [42], as well as epigenomic methylation analyses (EWAS) identifying beneficial alterations in CpG sites associated with aging [44].

Other CALERIE-derived studies have identified positive effects of CR on telomere length and the expression of genes associated with senescence [43], as well as associations between genetic variants (e.g., FTO) and reduced adherence to CR [45].

Despite its benefits, the clinical implementation of CR faces important limitations. Long-term adherence may be compromised without structured nutritional support, and losses of lean mass or bone density have been documented in specific subgroups [16,53]. Moreover, the effects on IGF-1 have proven inconsistent, likely influenced by factors such as age, sex, and dietary composition [13,16,30].

Within the field of caloric restriction, landmark clinical trials consistently demonstrate beneficial effects on cardiometabolic and epigenetic parameters (Table 2).

Taken together, the available evidence supports that a moderate, well-structured, and clinically supervised CR can induce metabolic, functional, and molecular improvements in humans, positioning it as a promising strategy in the context of healthy longevity.

### 3.2. Clinical Evidence of Intermittent Fasting (IF)

Intermittent fasting (IF) has emerged as an alternative dietary strategy to continuous caloric restriction (CR), based on the cyclical alternation between feeding and fasting periods, without necessarily requiring sustained caloric reduction. Among its most common modalities are alternate-day fasting (ADF), the 5:2 diet, and time-restricted feeding (TRF), such as the 16:8 protocol. Unlike classical CR, IF offers greater flexibility and may improve clinical adherence in certain populations [18,21].

At the molecular level, IF activates metabolic pathways shared with CR, including increased ketone body production, activation of AMPK and sirtuins, inhibition of mTOR, and stimulation of autophagic processes [13]. These adaptations have been associated with enhanced energy efficiency, reduced systemic inflammation, improved mitochondrial metabolism, and protection against oxidative damage [18,50].

Several clinical trials have documented the beneficial effects of IF on cardiometabolic parameters. Controlled studies have reported significant reductions in body weight, fasting glucose, blood pressure, and LDL cholesterol levels in overweight or prediabetic individuals [37]. A recent meta-analysis integrating more than 25 randomized trials concluded that IF significantly improves glucose, glycated hemoglobin (HbA1c), lipid profile, and blood pressure, with the most significant impact observed in individuals with baseline metabolic dysfunction [36].

In older adults, some interventions have demonstrated additional benefits, such as reductions in IL-6, improvements in cognitive function, and short-term preservation of lean mass [35]. A four-week randomized trial using an ADF protocol revealed increases in ketone bodies, reductions in glucose and systolic blood pressure, and upregulation of autophagy markers in healthy individuals [38].

Furthermore, IF may enhance its effects when aligned with circadian rhythms. In this regard, early time-restricted feeding (eTRF) has shown additional benefits on insulin sensitivity and glycaemic control when food intake is concentrated in the earlier hours of the day [19].

Despite these promising findings, practical limitations have been highlighted. Adherence may be compromised by work and social factors; moreover, prolonged protocols (>18 h of fasting) may induce hypoglycaemia, fatigue, or insomnia in older or frail individuals [13]. The long-term effects on bone density and muscle mass remain uncertain, and further studies in vulnerable populations are needed.

Regarding intermittent fasting, both clinical trials and reviews have reported metabolic and anti-inflammatory improvements, although with considerable methodological heterogeneity (Table 3).

Overall, IF represents a nutritional intervention with high clinical and translational potential, particularly in the context of cardiometabolic prevention and healthy aging. However, its application must be personalized according to age, chronotype, sex, and comorbidities.

### 3.3. Clinical Evidence of Caloric Restriction Mimetics (CR-Mimetics)

Caloric restriction mimetics (CR-mimetics) are compounds capable of inducing molecular adaptations in the organism like those observed with CR or intermittent fasting (IF), without the need to reduce total caloric intake. Their clinical relevance lies in their potential to activate longevity pathways, while offering greater dietary adherence and flexibility [31].

Among the most extensively studied are resveratrol, metformin, and rapamycin, due to their ability to modulate critical aging pathways, such as AMPK, mTOR, and SIRT1 (sirtuin-1), as well as to induce autophagy and reduce oxidative stress [10,13]. In addition, compounds such as spermidine, fisetin, acarbose, and several natural polyphenols have emerged as promising candidates with mimetic properties, exerting effects on autophagy, insulin sensitivity, and inflammation [51].

In clinical trials, resveratrol has demonstrated beneficial effects on blood pressure, insulin sensitivity, and SIRT1 expression in older adults, accompanied by improvements in antioxidant capacity [47]. Metformin, widely used in the treatment of type 2 diabetes, has been associated with cognitive improvements, reductions in inflammatory biomarkers (IL-6, TNF-α), and activation of autophagic processes, even in non-diabetic individuals [46,55]. Its impact on aging is currently being evaluated in the TAME (Targeting Aging with Metformin) clinical trial [32].

Rapamycin, a direct inhibitor of mTOR, has been the subject of several human trials, where it has shown improvements in glycemic control, reductions in IGF-1, and possible modulatory effects on the gut microbiota [52]. However, its adverse effect profile and risk of immunosuppression remain limiting factors for its widespread use in healthy populations.

A recent systematic review identified more than 20 controlled clinical trials evaluating different mimetics, including both dietary and pharmacological compounds. These studies reported positive effects on longevity biomarkers, including AMPK, SIRT1, IGF-1, HbA1c, serum lipids, telomere length, and DNA methylation [51].

In parallel, several CR mimetics, including metformin, resveratrol, and rapamycin, have demonstrated promising molecular and clinical effects (Table 4).

Nevertheless, CR-mimetics present important limitations: significant side effects (particularly with rapamycin), potential drug–drug interactions, and the scarcity of long-term trials in healthy individuals. Moreover, the extent to which these compounds fully replicate the systemic benefits of CR remains debated, and their effect on human longevity has not yet been conclusively demonstrated [10,18,21].

As a clinical strategy, mimetics could be incorporated into combined interventions that include personalized diet, physical exercise, and medical supervision, particularly in individuals with low adherence to classical dietary interventions.

### 3.4. Cross-Sectional Comparison of CR, IF, and CR-Mimetics

Although caloric restriction (CR), intermittent fasting (IF), and dietary mimetics share multiple molecular targets related to longevity, their clinical applications, sustainability, and risk–benefit profiles present substantial differences that condition their practical implementation.

Both CR and IF have demonstrated in humans the capacity to inhibit the mTOR pathway, activate AMPK and sirtuins, and significantly induce autophagy [13,18,21]. These pathways promote improvements in insulin sensitivity, reductions in oxidative stress, attenuation of inflammatory markers, and deceleration of epigenetic biomarkers of aging [16,23,42].

CR-mimetics, in turn, aim to reproduce these adaptations through pharmacological or dietary agents, such as resveratrol, metformin, rapamycin, or spermidine, with varying degrees of efficacy and clinical evidence [31,32,47]. Some of these compounds have shown positive effects on IGF-1, HbA1c, telomere length, DNA methylation, and autophagy in pilot studies and controlled clinical trials [43,52].

From a clinical perspective, CR is supported by robust and longitudinal trials, such as the CALERIE study; however, its widespread application is limited by poor adherence and adverse effects, including loss of bone or muscle mass [16,42]. By contrast, IF offers greater flexibility and social acceptance, with consistent benefits in body composition, glycemic profile, and inflammation, although with higher interindividual variability [21,50]. CR-mimetics represent a promising pharmacological alternative, but they still face safety challenges, a lack of long-term studies in healthy populations, and heterogeneity of results [10,47,52].

Table 5 presents a comparative synthesis of the primary molecular, clinical, and translational characteristics of these three strategies, facilitating the visualization of their convergent mechanisms and key differences in clinical applicability.

The three strategies share a common molecular core centered on the optimization of bioenergetics, protection against cellular damage, and epigenetic modulation of aging. However, their clinical choice must be tailored to the patient’s profile, preferences, expected adherence, and potential risks. A rational combination of dietary and pharmacological interventions may represent an effective and sustainable hybrid clinical model for preventing pathological aging.

The evidence synthesized in Table 1, Table 2, Table 3, Table 4 and Table 5 reflects the diversity of approaches and outcomes observed in humans and experimental models, providing a comparative framework to analyze the opportunities and limitations of each strategy. These findings form the basis for the critical discussion and translational proposal presented below.

## 4. Discussion

The clinical implementation of strategies such as CR, IF, and mimetics remains conditioned by the gap between molecular mechanisms and demonstrable functional benefits. The validation of biomarkers, such as epigenetic or transcriptomic clocks, is a promising step, but it is still insufficient. It will be essential to design trials that prioritize hard clinical endpoints, integrate personalized models, and evaluate sustainability in real-world healthcare settings.

### 4.1. Clinical Applicability and Translational Biomarkers

Although the molecular mechanisms underlying caloric restriction (CR), intermittent fasting (IF), and their mimetics are well-characterized in animal models, their clinical translation to humans remains one of the main limitations for systematic implementation [56]. This section critically evaluates such translation from four key perspectives: validity of mechanisms, available biomarkers, clinical endpoints, and structural barriers.

(a) Molecular mechanisms: limited extrapolation to humans

Studies in non-human primates and murine models have consistently shown that CR can extend lifespan, modulate pathways such as mTOR, SIRT1, and AMPK, improve autophagy, and delay the onset of chronic diseases [5,34,57]. However, in humans, these effects are attenuated or modified by several factors:

- Human baseline longevity is already high, precluding trials with direct survival endpoints.
- Comorbidities, polypharmacy, psychosocial environment, and genetic diversity complicate the reproduction of effects observed in animals [33].
- In older humans, IGF-1 inhibition—associated with longevity in mice—may be linked to muscle loss or frailty [58].

(b) Longevity biomarkers: utility and limits

Human studies have attempted to validate the effects of these interventions using intermediate biomarkers such as glucose, insulin, IGF-1, TNF-α, or C-reactive protein (CRP). While useful for metabolic monitoring, these markers do not directly correlate with functionality or years lived without disability [41].

In recent years, epigenetic clocks have been proposed as a promising tool for studying aging. Trials such as CALERIE II have shown that two years of CR reduce the pace of aging, as measured by the DunedinPACE algorithm, although without changes in other clocks, such as GrimAge or PhenoAge [33,57,59]. Additionally:

- Telomere length has yielded ambiguous results, with a possible accelerated loss in the early phases of CR intervention [60].
- Transcriptomic biomarkers derived from muscle biopsies have demonstrated changes in proteostasis, mitochondrial biogenesis, and apoptosis pathways, correlated with functional improvements [61].
- Composite measures of biological age (e.g., Klemera–Doubal, homeostatic dysregulation) have also shown slowing after CR, independent of weight loss [62].

(c) Clinical endpoints: an unmet need

To date, none of the strategies (CR, IF, or mimetics) have demonstrated benefits in clinically relevant endpoints such as:

- Reduction in hospitalizations or all-cause mortality.
- Sustained improvement in physical or cognitive function.
- Decrease in years lived with disability [41,63].

Most trials prioritize short-term metabolic outcomes without directly assessing parameters such as frailty, sarcopenia, immunosenescence, or resilience to physiological stress.

(d) Structural and contextual barriers

Even if mechanisms and biomarkers were validated, clinical applicability requires an adequate healthcare framework:

- Limited training in nutritional medicine, chronobiology, and geroscience among clinical professionals [64].
- Lack of structured tools for behavioral support, digital monitoring, or individualized biofeedback.
- Fragmented healthcare systems that hinder multidisciplinary approaches (nutritionists, psychologists, physicians, geriatricians).

(e) Emerging perspectives: integrated biomarkers and artificial intelligence for longevity medicine

In light of the current limitations in validating CR, IF, and mimetic interventions, integrative approaches are being developed that combine omics data (epigenetic, transcriptomic, metabolomic) with artificial intelligence (AI). These strategies enable the identification of complex biological patterns that predict individual responses to dietary and pharmacological interventions.

Advanced epigenetic algorithms (e.g., DunedinPACE, GrimAge2), combined with transcriptomic measures (FOXO3 pathways, proteostasis, mitochondrial biogenesis) and integrated metabolomics models, offer new composite metrics of biological aging. Their validation in trials such as CALERIE and DO-HEALTH enables not only the measurement of the molecular impact of interventions but also the personalization of strategies according to aging rate, inflammatory profile, or individual metabolic risk.

Integrating these metrics into digital health platforms, alongside predictive AI tools, opens up the possibility of designing personalized longevity interventions that can be monitored in real time, with adaptive feedback based on dynamic biomarkers. This translational approach constitutes one of the most promising avenues for bringing longevity medicine from the laboratory to the patient.

As summarized in Table 5, the three strategies exhibit consistent evidence, albeit with varying degrees of robustness. At the molecular level, both CR and IF have repeatedly demonstrated the activation of AMPK and sirtuins, inhibition of mTOR, and stimulation of autophagy in animal models [33,34,56,58]. Mimetics partially reproduce these pathways, although efficacy depends on the compound and dose [33,41,59].

Regarding epigenetic markers, CR has shown reductions in biological age, as measured by DunedinPACE in CALERIE, but no clear changes in GrimAge or PhenoAge [41,58]. If data are mixed and inconclusive, while some CR-mimetics, such as resveratrol or metformin, have shown modest modulations, they still lack robust evidence in humans [33].

In the fields of transcriptomics and metabolomics, CR modifies the expression of genes involved in circadian rhythms, proteostasis, and mitochondrial biogenesis [41], in addition to reducing biological age according to metabolic indices, such as those proposed by Klemera and Doubal. In IF, changes are mainly limited to glucose and insulin [33], while in CR-mimetics research remains at an early stage, with hypotheses regarding effects on microbiota-derived metabolites and insulin pathways [33,59].

Clinical biomarkers reinforce the utility of CR and IF, with reductions in IGF-1, insulin, CRP, and TNF-α in various trials [5,41,58], while CR-mimetics (metformin, resveratrol, rapamycin) are associated with improvements in HbA1c, IL-6, or mTOR activity [33,41,59]. However, in all cases, effects on telomere length remain inconsistent. With respect to clinical endpoints, neither CR, IF, nor CR mimetics have yet demonstrated reductions in mortality, disability, or major clinical events [33,41,58]. However, partial improvements in quality of life or intermediate functional parameters have been observed [41].

**Figure 2:**
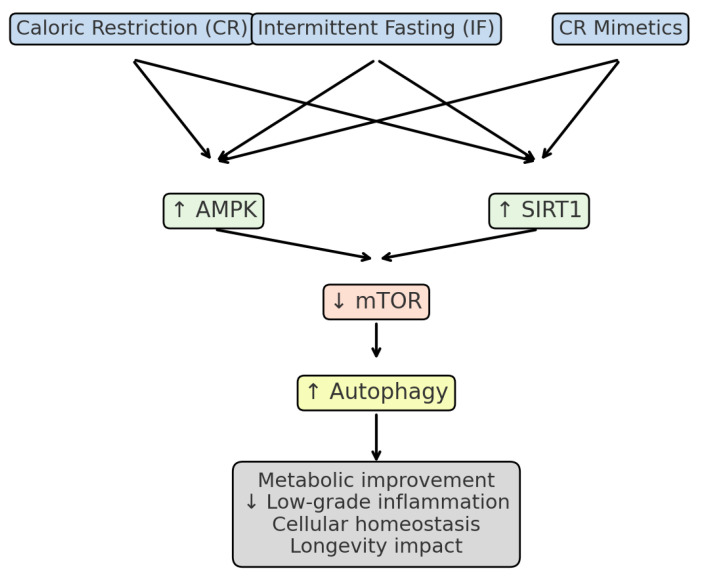
Schematic representation of the main molecular pathways modulated by caloric restriction (CR), intermittent fasting (IF), and CR mimetics. These interventions converge on AMPK and SIRT1 activation, as well as mTOR inhibition, promoting autophagy, improved metabolic efficiency, and reduced low-grade inflammation, all of which are associated with healthier aging trajectories. This figure provides a simplified overview of the best-established molecular interactions; other regulators (e.g., FOXO transcription factors, mitochondrial biogenesis) also contribute but are not shown for clarity.

To facilitate comprehension, Figure 2 provides a simplified schematic of the main molecular pathways modulated by CR, IF, and CR mimetics, highlighting AMPK and SIRT1 activation, mTOR inhibition, and the induction of autophagy

Finally, regarding implementation, prolonged CR is associated with poor adherence and a risk of lean mass loss [57]; IF, although more socially accepted, shows heterogeneous individual responses and possible hormonal alterations [60,61]; while CR-mimetics are easier to administer but lack long-term safety studies and behavioral support components [33,41,59].

Taken together, these differences condition translational applicability and highlight the need for an adaptive model such as AMAL.

### 4.2. Current Gaps and Future Directions

Despite significant growth in knowledge on dietary–metabolic strategies for longevity, the field still faces structural limitations that hinder clinical validation and real-world application in diverse populations. This section identifies the main methodological, physiological, and translational gaps that must be addressed, as well as the priority lines of research required for effective development.

(a) Insufficient clinical trials: duration, sample size, and endpoints

A significant proportion of current trials on caloric restriction (CR), intermittent fasting (IF), and energy mimetics:

- Have a duration ≤12 months, which prevents the evaluation of clinically relevant outcomes such as frailty, sustained physical function, major cardiovascular events, or healthy longevity.
- Focused on particular populations (young healthy adults), with insufficient representation of:
  – Adults over 70 years,
  – Individuals with comorbidities or polypharmacy,
  – Subjects in socioeconomically or ethnically vulnerable contexts.
- Assess intermediate outcomes (biomarkers) instead of hard clinical outcomes, such as mortality reduction, functional decline, or loss of independence.

The scientific community calls for a new generation of multicenter trials, stratified by frailty, lasting at least 3–5 years, and incorporating objective functional and clinical endpoints [65,66,67].

(b) Limited integration of multidimensional biomarkers

Most studies rely on conventional biomarkers (such as glucose, lipids, insulin, and IL-6), which do not adequately capture the complexity of biological aging. A broader and deeper evaluation is needed, including:

- Validated epigenetic clocks (DunedinPACE, PhenoAge, GrimAge) as primary outcomes.
- Transcriptomic, metabolomic, and proteomic profiling to characterize responders vs. non-responders.
- Immunological cluster analyses linked to cellular senescence, immune resilience, and functional capacity.
- Longitudinal assessment of gut microbiota with indicators such as the Firmicutes/Bacteroidetes ratio, SCFA production, alpha diversity, and abundance of *Akkermansia muciniphila* [58,68,69].

(c) Insufficient personalization: from population average to clinical phenotype

Current strategies are broadly applied homogeneously, without accounting for patient variability in metabolism, chronobiology, hormones, genetics, or microbiome. To enhance efficacy, the following are required:

- Phenotypic classification by chronotype, inflammatory pattern, anabolic resistance, and microbiota profile.
- Adaptive and dynamic protocols, adjusted according to clinical evolution and treatment response.
- Predictive tools based on artificial intelligence and machine learning, integrated into the electronic health record [70,71].

(d) Adherence: the underestimated barrier

Despite its clinical relevance, adherence to restrictive dietary interventions has been poorly evaluated in reviewed studies. Fewer than 40% of trials systematically monitor this parameter. Future strategies should include:

- Mobile applications with real-time feedback, wearable sensors (glucose, HRV, physical activity), and individualized remote monitoring.
- Behavioral motivation techniques (MI), positive reinforcement, and nutritional coaching with psychosocial support.
- Predictive adherence tools based on clinical, psychological, and social data, supported by AI and predictive modeling [72,73].

(e) Clinical implementation and healthcare sustainability

For these strategies to yield real clinical benefits, they must be integrated into healthcare systems through:

- Cost-effectiveness evaluation of nutritional and metabolic longevity programs.
- Specific training of healthcare professionals in longevity medicine, microbiota, and applied AI.
- Development of clinical algorithms and biomedical panels integrated into electronic health record platforms to personalize prescription and follow-up [73,74].

Dietary strategies targeting longevity have demonstrated biological and clinical potential; however, their widespread implementation requires overcoming critical limitations, including the need for higher-quality trials, robust biomarkers, personalized models, sustained adherence strategies, and functional clinical infrastructures. Only then will it be possible to translate mechanistic advances into effective and scalable preventive medicine.

### 4.3. Clinical–Translational Proposal: Rationale and Justification

One of the main challenges in implementing dietary metabolic strategies in old age lies in their real clinical applicability. Although evidence on CR, IF, and molecular mimetics is increasingly solid in experimental models and pilot studies, their effective translation into medical practice is limited by patient heterogeneity, comorbidity burden, variable adherence, and the lack of dynamic personalization tools [65,66].

Here, we propose the Active Management of Aging and Longevity (AMAL) model, a clinical–translational framework designed to actively integrate dietary, metabolic, and lifestyle strategies for the proactive management of aging processes and the extension of healthy life. It is structured into three progressive levels based on the individual’s functional, metabolic, and behavioral profile. This proposal is grounded in consolidated mechanistic principles, advanced assessment tools, and criteria of healthcare feasibility.

Level 1: Personalized basal intervention (primary prevention)

Targeted to young or middle-aged adults with low metabolic risk and high autonomy, this level focuses on low metabolic impact but high adherence interventions. Recommended strategies include circadian IF (16:8) or mild CR (10–15%), complemented by nutritional education, chrononutrition, multicomponent exercise, and digital monitoring tools [18,50,75].

Level 2: Combined bioactive intervention (secondary prevention)

For individuals with metabolic syndrome, insulin resistance, or subclinical inflammation without frailty, this level proposes a combination of moderate CR (15–20%), IF protocols adapted to chronotype, functional foods rich in polyphenols, and, in selected cases, mimetics such as metformin or resveratrol. Monitoring of hepatic/renal parameters, protein balance, and inflammation markers (CRP, IL-6) is recommended during follow-up [32,76].

Level 3: Advanced personalized intervention (tertiary prevention/clinical longevity)

Indicated for older, frail patients with polypharmacy or complex chronic diseases. Here, the priority is to preserve functionality and minimize the risk of adverse events. Personalization is guided by biomarkers (epigenetic clocks such as GrimAge or DunedinPACE), gut microbiota analysis (alpha diversity, SCFAs, *Akkermansia*), inflammatory profile, and chronobiology. Aggressive caloric restriction is avoided, favoring dietary–pharmacological modulation, interdisciplinary support, and technological decision-support tools [58,69,70,71].

Operational deployment

The clinical implementation of AMAL requires a support structure that integrates traditional tools (such as glucose, insulin, lipid profile, IGF-1, and frailty tests) with molecular panels and digital platforms (including AI, apps, and wearables). Clinical decisions should be based on predictive algorithms that combine clinical, biochemical, and behavioral data in real time [41,71].

Applied examples

- A 78-year-old man with frailty, elevated IL-6, and microbiota poor in butyrate may benefit from an anti-inflammatory diet with mild IF, resveratrol supplementation, and weekly telemonitoring support.
- A 36-year-old woman with HPA axis dysfunction and high estrogen sensitivity may require extended fasting windows, psychological support, and hormonal adjustments without strict CR [77].

This progressive and adaptive framework enables integration of CR, IF, and mimetics into real clinical scenarios, maximizing impact without compromising safety or adherence. Although prospective validation is still ongoing, the AMAL model provides a solid foundation for building evidence-based longevity medicine. The AMAL framework is presented here as a conceptual model. Defining specific dosing protocols, intervention durations, and monitoring parameters for CR mimetics will require further clinical trials and lies beyond the scope of this review.

With the AMAL framework defined, it is pertinent to critically integrate the evidence presented in the synthesis (Table 1, Table 2, Table 3, Table 4 and Table 5) and the identified gaps to comparatively position CR, IF, and mimetics, highlight their mechanistic convergences (↓ IGF-1, ↑ AMPK/SIRT1, ↓ mTOR, ↑ autophagy), and delineate their current clinical applicability.

### 4.4. Integrative Synthesis

Dietary strategies that modulate biological aging, such as CR, IF, and molecular mimetics, have gained prominence not only as longevity tools but also as metabolic interventions with high clinical potential. This review has compiled and compared relevant human evidence on these strategies, emphasizing their standard molecular mechanisms—such as mTOR inhibition, AMPK/SIRT1 activation, and autophagy stimulation—and their impact on cardiometabolic, inflammatory, and epigenetic biomarkers.

One of the most consistent findings is the convergence of molecular pathways activated by CR and IF. Both strategies induce a hypocatabolic metabolic state that favors AMPK activation and mTOR inhibition, thereby facilitating cellular autophagy—especially mitophagy—and promoting metabolic resilience against oxidative stress [39,40]. These effects are not merely theoretical: human studies, such as CALERIE, have demonstrated sustained improvements in insulin sensitivity, blood pressure, and CRP, as well as significant reductions in the pace of epigenetic aging, measured by clocks such as DunedinPACE [48,58].

IF—particularly in its 16:8 modality or early time-restricted feeding—has shown efficacy comparable to CR in improving glycaemic control, blood pressure, and lipid profiles, with better tolerance and adherence in most studies [49]. Mattson and colleagues have been especially influential in establishing the benefits of IF on neuroprotection, HPA axis modulation, and synaptic plasticity, broadening its potential impact beyond classical cardiometabolic pathways [78].

CR-mimetics offer an attractive pharmacological avenue, although they are still in the consolidation phase. Compounds such as metformin, rapamycin, resveratrol, and spermidine have shown effects on pathways similar to those activated by CR/IF, including improvements in autophagy, IGF-1, inflammation, and lipid profile [31,32]. Trials, such as those by Mannick et al. using mTOR inhibitors in older adults, have reported promising immunomodulatory effects. However, concerns remain regarding long-term safety and applicability beyond specific populations [76].

Despite these advances, significant gaps in evidence persist. Many trials last ≤12 months include homogeneous populations (young, healthy adults), and do not employ hard clinical endpoints (frailty, hospitalizations, functional decline) [79]. Moreover, few studies integrate molecular biomarkers of biological aging as primary outcomes. The lack of tools to distinguish responders from non-responders hinders refinement of interventions, while adherence remains a neglected factor in numerous trials.

Another critical aspect is clinical personalization. Interindividual variability in chronotype, microbiota, inflammatory profile, hormonal sensitivity, and anabolic resilience strongly influence the effectiveness of these strategies. As demonstrated by the AMAL model proposed in this work, only a personalized approach—supported by biomarkers, clinical phenotypes, and digital tools—can translate the potential of CR, IF, and mimetics into viable, scalable precision metabolic medicine [71].

Beyond dietary interventions and CR mimetics, recent pharmacological advances—particularly GLP-1 receptor agonists—have shown auspicious effects on glycemic control and body weight, accompanied by reductions in markers of low-grade inflammation. These findings suggest that GLP-1 agonists may represent a valuable complementary strategy for the future, especially in combination with dietary approaches such as CR and IF. Their integration into precision medicine frameworks could be a logical next step to optimize metabolic modulation and translational impact on longevity [71].

From a public health and preventive perspective, these interventions hold significant implications for conditions such as metabolic syndrome, type 2 diabetes, sarcopenic obesity, and even early cognitive decline. Integrating these strategies into real-world clinical programs requires implementation trials, interdisciplinary team training, and digital tools that enable professionals to modulate interventions according to patient profiles.

In summary, CR, IF, and their mimetics represent not only longevity strategies but also therapeutic tools against metabolic aging. Their clinical integration will depend on biomarker validation, protocol personalization, and healthcare system capacity to adopt a proactive, predictive, and adaptive medicine approach. This transformation is both achievable and urgent.

Accordingly, it is necessary to analyze separately how CR, IF, and mimetics impact highly prevalent clinical conditions such as type 2 diabetes, non-alcoholic fatty liver disease (NAFLD), and metabolic syndrome (MetS). This organization allows tracing, in each case, the full axis from implicated molecular mechanisms to intermediate biomarkers and clinical outcomes, reinforcing the translational relevance of the reviewed strategies.

#### 4.4.1. Type 2 Diabetes (T2D)

Type 2 diabetes (T2D) is the most studied clinical model at the intersection of metabolic aging and energy-restriction strategies. Both CR and IF modulate central pathways, such as AMPK and SIRT1, which secondarily reduce mTOR signaling and activate autophagy [31,32]. At the biomarker level, consistent reductions have been observed in fasting glucose, insulin, HOMA-IR, and HbA1c, along with improvements in inflammatory markers such as IL-6 and CRP [41,60]. Mimetics, particularly metformin, have shown reductions in HbA1c and improvements in insulin sensitivity in patients with T2D [31]. However, human trials remain focused on intermediate outcomes, without yet demonstrating reductions in major complications or mortality.

#### 4.4.2. Non-Alcoholic Fatty Liver Disease (NAFLD)

NAFLD is closely associated with insulin resistance and sarcopenic obesity and represents a key scenario to evaluate the clinical applicability of CR and IF. Mechanistically, AMPK activation and mTOR inhibition reduce hepatic lipogenesis and promote fatty acid oxidation [33,34,56,58]. Clinical trials have shown that CR significantly reduces liver fat and hepatic enzymes (ALT, AST), even in non-obese adults [16] (Redman 2018). IF protocols, such as 5:2 or time-restricted feeding, have shown reductions in hepatic triglycerides and improvements in insulin sensitivity [33]. Mimetics, such as resveratrol, have demonstrated hepatoprotective potential through SIRT1 activation and reduction in inflammation [33]. Despite these advances, clinical evidence remains limited in duration, and robust data on progression to steatohepatitis or cirrhosis are lacking.

#### 4.4.3. Metabolic Syndrome (MetS)

MetS integrates central obesity, dyslipidaemia, hypertension, and insulin resistance, thus encompassing the main cardiometabolic risk factors of aging. In this context, CR and IF act by reducing energy excess and modulating cellular stress pathways, resulting in consistent improvements in lipid profile, glucose levels, blood pressure, and systemic inflammation [57,59,60,61]. The most frequent biomarkers include reductions in triglycerides, LDL cholesterol, and CRP, along with increases in HDL cholesterol [33]. In older adults with MetS, IF protocols have been shown to improve both metabolic parameters and markers of inflammation, as well as cognitive performance [49]. CR-mimetics, such as rapamycin or berberine, exhibit preliminary glucose and lipid-regulating effects, although clinical evidence is limited [33,59].

Taken together, the available evidence shows that while CR, IF, and their mimetics converge on common biological mechanisms, their clinical effects vary according to disease and target population. This disease-specific analysis underscores both the opportunities and the persisting knowledge gaps, reinforcing the need for longer, personalized trials. The AMAL model is proposed as an integrative tool capable of translating these findings into adaptive clinical protocols, aimed at prevention and active management of major metabolic diseases associated with aging.

**Figure 3:**
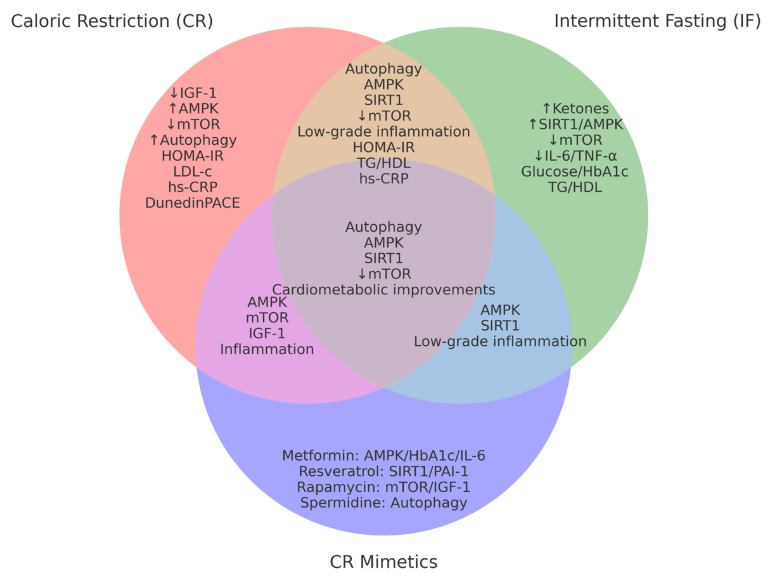
Venn diagram summarizing shared and specific mechanisms/biomarkers across caloric restriction (CR), intermittent fasting (IF), and CR mimetics. Overlapping regions highlight convergent pathways (AMPK/SIRT1 activation, mTOR inhibition, autophagy, reduction in low-grade inflammation). Non-overlapping areas illustrate mechanisms more consistently associated with each intervention (e.g., ↓ decreased IGF-1 with CR, ↑ increased ketones with IF, compound-specific effects with mimetics). This schematic provides a visual synthesis complementing the detailed evidence reported in Table 1.

A visual synthesis of these findings is provided in Figure 3, which highlights shared and specific mechanisms across CR, IF, and CR mimetics.

## 5. Conclusions

Caloric restriction (CR), intermittent fasting (IF), and caloric restriction mimetics (CR-mimetics) represent promising strategies for modulating biological aging and improving cardiometabolic parameters. Available clinical evidence is consistent in intermediate biomarkers, particularly in type 2 diabetes, NAFLD, and metabolic syndrome, but remains insufficient regarding hard clinical outcomes and long-term studies. The integration of advanced biomarkers and the personalization of protocols emerge as essential requirements to translate these approaches into clinical practice. The AMAL model offers a progressive and adaptable framework to guide this translation, while remaining a conceptual proposal pending clinical validation and the development of dedicated digital tools.

## Figures and Tables

**Table 1:** Studies included in the integrative critical review were classified by intervention, study design, and main findings.

Author(s)	Brief Title	Main Topic	Study Type	Key Finding or Relevance	Year
Kennedy BK et al. [10]	Geroscience link to chronic disease	Conceptual aging framework	Review	Establishes aging as the root of chronic diseases	2014
[5]	Molecular hallmarks of aging	Aging biology	Review	Defines nine molecular hallmarks of aging	2013
[30]	Hallmarks of health	Health and resilience pathways	Review	Extends the model to health-protective mechanisms	2021
[31]	CR mimetics	Molecular targets, therapy	Review	Spermidine, resveratrol, and others as mimetics	2019
[13]	CR mechanisms across species	Comparative metabolism	Review	Differences in CR impact by species	2022
[32]	CR and mimetics	Integrated perspective on longevity	Review	Combines metabolic and molecular insights	2021
[19]	Chrononutrition	Circadian–metabolic interaction	Animal study	Diet rhythm modulates metabolic pathways	2017
[33]	Epigenetic clocks	Biomarkers of aging	Clinical study	GrimAge validated for lifespan and healthspan	2018
[34]	Caloric restriction	Non-human primate longevity	Experimental study	CR improves survival and function in monkeys	2017
[31]	Fasting-mimicking diet	Periodic restriction	Clinical trial	FMD improves IGF-1, glucose, regeneration	2019
[35]	Intermittent fasting	Metabolic reprogramming	Review	Switch to fat oxidation, metabolic flexibility	2018
[36]	IF and metabolic markers	Cardiometabolic health	Review	IF improves insulin, glucose, and lipids	2024
[16]	CR and NAFLD	Visceral fat and liver	Clinical trial	CR reduces hepatic fat in non-obese adults	2018
[37]	Alternate-day fasting	Body composition	Clinical trial	ADF reduces fat mass and improves lipids	2020
[19]	Circadian IF	Chronobiology and metabolism	Review	Aligning meals to rhythms boosts IF effects	2017
[38]	Prolonged fasting	Safety and tolerability	Human observational	Safe in a large cohort with improved well-being	2019
[39]	CR translation across species	Translational medicine	Review	Bridges preclinical and human evidence	2015
[40]	Metabolic control of longevity	Mitochondrial networks	Review	Metabolism is central to lifespan modulation	2016
[41]	CR and epigenetics	DNA methylation clocks	RCT (CALERIE)	CR slows epigenetic aging (PhenoAge, GrimAge)	2023
[42]	CR and transcriptomics	Muscle stress and longevity genes	RCT (CALERIE)	CR shifts gene expression toward resilience	2023
[43]	CR and telomere biology	Cellular senescence	RCT (CALERIE)	CR preserves telomere length	2024
[44]	CR and EWAS	Epigenomic modulation	RCT (CALERIE)	CR alters aging-related CpG methylation	2022
[23]	CR and biological pace	DunedinPACE biomarker	RCT (CALERIE)	CR slows the molecular aging rate	2017
[45]	FTO polymorphism and CR	Genetic determinants of adherence	RCT (CALERIE)	FTO SNPs linked to lower CR adherence	2021
[46]	Metformin and cognition	Cognitive performance in T2D	RCT	Improves memory, linked to HbA1c drop	2014
[47]	Resveratrol in aging adults	SIRT1 and oxidative stress	RCT	↑ SIRT1, antioxidant capacity in the elderly	2023
[36]	Intermittent fasting meta-review	Health outcomes	Umbrella review	Consistent benefits on glucose, weight, and lipids	2024

Abbreviations: CR, caloric restriction; IF, intermittent fasting; FMD, fasting-mimicking diet; NAFLD, non-alcoholic fatty liver disease; RCT, randomized controlled trial; IGF-1, insulin-like growth factor 1; SNP, single-nucleotide polymorphism; HbA1c, glycated hemoglobin.

**Table 2:** Key clinical trials on caloric restriction (CR).

Study/Author	Design/Sample	Duration	Biomarkers Evaluated	Main Findings
[48]	RCT, 218 adults, 25% CR	24 months	Weight, glucose, insulin, CRP, IGF-1	Reduced weight, inflammation, and improved insulin sensitivity
[23]	CALERIE epigenetic substudy, 197 participants	24 months	Epigenetic clocks	Slowed epigenetic aging (~2–3%)
[45]	CALERIE follow-up, 105 participants	6–12 months	Glucose, lipids, and insulin sensitivity	Maintained cardiometabolic benefits

CR: caloric restriction; RCT: randomized controlled trial; CRP: C-reactive protein; IGF-1: insulin-like growth factor 1.

**Table 3:** Key clinical trials on intermittent fasting (IF).

Study/Author	Design/Sample	Duration	Biomarkers Evaluated	Main Findings
[49]	RCT, 116 overweight adults, 16:8 regimen	12 weeks	Weight, glucose, insulin, BP	Weight loss; no major insulin changes
[25]	RCT, obese adults, 5:2 vs. IF	12 weeks	BMI, lipids, glucose	Reduced fat mass, improved cardiometabolic markers
[50]	Trial, older adults with metabolic syndrome	8 weeks	CRP, IL-6, TNFα, glucose	Improved inflammation and metabolic profile
[36]	Systematic review of 25 RCTs	4–52 weeks	Glucose, HbA1c, cholesterol, BP	IF improves metabolic markers

IF: intermittent fasting; CR: caloric restriction; RCT: randomized controlled trial; CRP: C-reactive protein; IL-6: interleukin-6; TNFα: tumor necrosis factor alpha; BP: blood pressure.

**Table 4:** Key clinical trials on CR mimetics.

Study/Author	Design/Sample	Duration	Biomarkers Evaluated	Main Findings
[51]	RCT, 124 adults, placebo-controlled, oral resveratrol	6 months	BP, TAC, GPx, SH/GSSG, TG, cholesterol, HOMA-IR, SIRT1, insulin, glucose	Resveratrol improved SIRT1, SIRT1, TAC, GPx, ↓ TG; no significant change in BP
[52]	RCT, older adults, metformin vs. placebo	6 months	IL-6, TNFα, glucose, cognition	Metformin reduced inflammation, improved metabolism
[51]	RCT, prediabetes patients, rapamycin	10 weeks	IGF-1, mTOR, HbA1c, microbiota	Rapamycin reduced IGF-1, improved insulin sensitivity
[51]	Systematic review of 18 studies	8–52 weeks	AMPK, mTOR, sirtuins, glucose, lipids	Mimetics replicate CR molecular effects

CRM: caloric restriction mimetic; RCT: randomized controlled trial; BP: blood pressure; IL-6: interleukin-6; TNFα: tumor necrosis factor alpha; IGF-1: insulin-like growth factor 1; mTOR: mechanistic target of rapamycin; AMPK: AMP-activated protein kinase; TAC: total antioxidant capacity; GPx: glutathione peroxidase; TG: triglycerides; HOMA-IR: homeostatic model assessment of insulin resistance.

**Table 5:** Comparative clinical and molecular effects of CR, IF, and CR mimetics.

Characteristic	CR (Caloric Restriction)	IF (Intermittent Fasting)	CR Mimetics
Intervention type	Continuous caloric reduction	Restricted feeding windows	Use of compounds activating longevity pathways
Main mechanisms	↓ IGF-1, ↑ AMPK, ↓ mTOR, ↑ autophagy	↑ Ketone bodies, ↑ SIRT1, ↑ AMPK, ↓ mTOR	↑ SIRT1, ↓ mTOR, ↑ autophagy, ↓ inflammation
Duration in trials	6–24 months	8–12 weeks	8–24 weeks (pilot trials)
Biomarkers evaluated	IGF-1, CRP, glucose, DNA methylation	Glucose, IL-6, ketones, TNF-α	AMPK, IGF-1, HbA1c, autophagy, epigenetics
Clinical advantages	High efficacy, strong evidence base	Well tolerated, adaptable	Potential pharmacological application
Limitations	Low adherence, lean mass loss risk	Variable adherence, heterogeneous effects	Side effects, lack of long-term studies
Translational applicability	High (requires clinical supervision)	High (personalized by chronotype, age)	Moderate (under clinical research)

Abbreviations: CR, caloric restriction; IF, intermittent fasting; IGF-1, insulin-like growth factor 1; AMPK, AMP-activated protein kinase; mTOR, mechanistic target of rapamycin; SIRT1, sirtuin 1; CRP, C-reactive protein; HbA1c, glycated hemoglobin; IL-6, interleukin-6; TNF-α, tumor necrosis factor alpha.

## Data Availability

The original contributions presented in this study are included in the article/Supplementary Material. Further inquiries can be directed to the corresponding author(s).

## Supplementary Materials

The following supporting information can be downloaded at: https://www.mdpi.com/article/10.3390/ijms26199643/s1.

## Abbreviations

The following abbreviations are used in this manuscript:

ADF	Alternate-Day Fasting
AMAL	Active Management of Aging and Longevity
AMPK	AMP-Activated Protein Kinase
BP	Blood Pressure
CR	Caloric Restriction
CRM	Caloric Restriction Mimetics
CRP	C-Reactive Protein
FMD	Fasting-Mimicking Diet
FTO	Fat Mass and Obesity-Associated Gene
HPA	Hypothalamic–Pituitary–Adrenal Axis
IF	Intermittent Fasting
IGF	Insulin-like Growth Factor
IL	Interleukin
LDL	Low-Density Lipoprotein
NAFLD	Non-Alcoholic Fatty Liver Disease
OR	Odds Ratio
RCT	Randomized Controlled Trial
TNF	Tumor Necrosis Factor
