# Supplementary material for: Dietary and Pharmacological Modulation of Aging-Related Metabolic Pathways: Molecular Insights, Clinical Evidence, and a Translational Model
Source: Int J Mol Sci. 2025 Oct 2;26(19):9643. doi: 10.3390/ijms26199643 (PMC12525316; doi:10.3390/ijms26199643)
Supplement: Supplementary file 1 [file ijms-26-09643-s001.zip › ijms-3899895-supplementary.pdf]

Table S1. Studies included in the review: characteristics, design, population, duration, biomarkers and main findings.

Author(s)	Brief Title	Main Topic	Study Type	Key Finding or Relevance	Year
López-Otín C, Blasco MA, Partridge L, Serrano M, Kroemer G [5]	Hallmarks of aging: An expanding universe	Aging / biomarkers (general)	Review	Scholarly review on Aging / biomarkers (general), mechanisms and implications.	2023
Barzilai N, Ferrucci L [6]	Insulin resistance and aging: a cause or a protective response? J Gerontol A Biol Sci Med Sci	Aging / biomarkers (general)	Review	Scholarly review on Aging / biomarkers (general), mechanisms and implications.	2012
Ingram DK, Roth GS [7]	Calorie restriction mimetics: can you have your cake and eat it, too? Ageing Res Rev	Aging / biomarkers (general)	Review	Scholarly review on Aging / biomarkers (general), mechanisms and implications.	2015
Guan L, Liu R [8]	The Role of Diet and Gut Microbiota Interactions in Metabolic Homeostasis	Aging / biomarkers (general)	Review	Scholarly review on Aging / biomarkers (general), mechanisms and implications.	2023
Franceschi C, Garagnani P, Parini P, Giuliani C, Santoro A [9]	Inflammaging: a new immune-metabolic viewpoint for	Aging / biomarkers (general)	Review	Scholarly review on Aging / biomarkers (general), mechanisms and implications.	2018

	age-related diseases				
Duan H, Pan J, Guo M, Li J, Yu L, Fan L [11]	Dietary strategies with anti-aging potential: Dietary patterns and supplements	Aging / biomarkers (general)	Review	Scholarly review on Aging / biomarkers (general), mechanisms and implications.	2022
Green CL, Lamming DW, Fontana L [13]	Molecular mechanisms of dietary restriction promoting health and longevity	Caloric restriction	Review	Scholarly review on Caloric restriction, mechanisms and implications.	2022
Weiss EP, Racette SB, Villareal DT, Fontana L, Steger-May K, Schechtman KB, Klein S, Holloszy JO; Washington University School of Medicine CALERIE Group [15]	Improvements in glucose tolerance and insulin action induced by increasing energy expenditure or de…	Aging / biomarkers (general)	Clinical trial (randomized/controlled)	Human clinical trial (randomized/controlled) evaluating Aging / biomarkers (general) and metabolic/aging outcomes.	2006
Most J, Gilmore LA, Smith SR, Han H, Ravussin E, Redman LM [16]	Significant improvement in cardiometabolic health in healthy nonobese	Caloric restriction	Review	Scholarly review on Caloric restriction, mechanisms and implications.	2018

	individuals during caloric re…				
Martin CK, Bhapkar M, Pittas AG, Pieper CF, Das SK, Williamson DA, Scott T, Redman LM, Stein R, Gilhooly CH, Stewart T, Robinson L, Roberts SB; Comprehensive Assessment of Long-term Effects of Reducing Intake of Energy (CALERIE) Phase 2 Study Group [17]	Effect of Calorie Restriction on Mood, Quality of Life, Sleep, and Sexual Function in Healthy Nonob…	Aging / biomarkers (general)	Clinical trial (randomized/controlle d)	Human clinical trial (randomized/controlled) evaluating Aging / biomarkers (general) and metabolic/aging outcomes.	2016
Longo VD, Panda S [18]	Fasting, Circadian Rhythms, and Time-Restricted Feeding in Healthy Lifespan	Intermittent fasting	Review	Scholarly review on Intermittent fasting, mechanisms and implications.	2016
Manoogian ENC, Panda S [19]	Circadian rhythms, time-restricted feeding, and healthy aging	Intermittent fasting	Review	Scholarly review on Intermittent fasting, mechanisms and implications.	2017

Kahleova H, Lloren JI, Mashchak A, Hill M, Fraser GE [20]	Meal Frequency and Timing Are Associated with Changes in Body Mass Index in Adventist Health Study 2	Aging / biomarkers (general)	Review	Scholarly review on Aging / biomarkers (general), mechanisms and implications.	2017
Mattson MP, Longo VD, Harvie M [21]	Impact of intermittent fasting on health and disease processes	Intermittent fasting	Review	Scholarly review on Intermittent fasting, mechanisms and implications.	2017
Dorn GW II [22]	Evolving Concepts of Mitochondrial Dynamics	Aging / biomarkers (general)	Review	Scholarly review on Aging / biomarkers (general), mechanisms and implications.	2019
Waziry R, Ryan CP, Corcoran DL, Huffman KM, Kobor MS, Kothari M, Graf GH, Kraus VB, Kraus WE.; et al [23]	Effect of long-term caloric restriction on DNA methylation measures of biological aging in healthy adults from the CALERIE trial	Caloric restriction	Clinical trial (randomized/controlled)	Human clinical trial (randomized/controlled) evaluating Caloric restriction and metabolic/aging outcomes.	2023
Chen YE, Tsai HL, Tu YK, Chen LW [24]	Effects of different types of intermittent	Intermittent fasting	Systematic review / meta-analysis	Evidence synthesis on Intermittent fasting with mechanistic/clinical focus.	2024

	fasting on metabolic outcomes: an umbrella review and ne…				
Alfahl SO [25]	Evaluation of the effectiveness of intermittent fasting versus caloric restriction in weight loss a…	Intermittent fasting	Systematic review / meta-analysis	Evidence synthesis on Intermittent fasting with mechanistic/clinical focus.	2025
Belsky, D.W.; Caspi, A.; Arseneault, L.; Baccarelli, A.; Corcoran, D.L.; Gao, X.; Hannon, E.; Harrington, H.L.; Rasmussen, L.J.H.; Houts, R.; et al [28]	Quantification of the pace of biological aging in humans through a blood test, the DunedinPoAm DNA methylation algorithm …	Aging / biomarkers	Review	Scholarly review on Aging / biomarkers (general), mechanisms and implications.	2020
Surendran P, Stewart ID, Au Yeung VPW, Pietzner M, Raffler J, Wörheide MA, Li C, Smith RF, Wittemans LBL, Bomba L, Menni C, et al. [29]	Rare and common genetic determinants of metabolic individuality and their effects on human health	Aging / biomarkers (general)	Review	Scholarly review on Aging / biomarkers (general), mechanisms and implications.	2022

Ros M, Carrascosa JM [53]	Current nutritional and pharmacological anti-aging interventions	Aging / biomarkers (general)	Review	Scholarly review on Aging / biomarkers (general), mechanisms and implications.	2020
Das JK, Banskota N, Candia J, Griswold ME, Orenduff M, de Cabo R, Corcoran DL, Das SK, De S, Huffman KM, Kraus VB, Kraus WE, Martin CK, Racette SB, Redman LM, Schilling B, Belsky DW, Ferrucci L [42]	Calorie restriction modulates the transcription of genes related to stress response and longevity i…	Aging / biomarkers (general)	Review	Scholarly review on Aging / biomarkers (general), mechanisms and implications.	2023
Ramaker ME, Corcoran DL, Apsley AT, Kobor MS, Kraus VB, Kraus WE, Lin DTS, Orenduff MC, Pieper CF, Waziry R, Huffman KM, Belsky DW [44]	Epigenome-wide Association Study Analysis of Calorie Restriction in Humans, CALERIE™ Trial Analysis	Aging / biomarkers (general)	Clinical trial (randomized/controlled)	Human clinical trial (randomized/controlled) evaluating Aging / biomarkers (general) and metabolic/aging outcomes.	2022
Hastings WJ, Ye Q, Wolf SE, Ryan CP, Das SK, Huffman KM, Kobor MS, Kraus WE, MacIsaac JL, Martin CK, Racette SB,	Effect of long-term caloric restriction on telomere length in healthy adults:	Caloric restriction	Clinical trial (randomized/controlled)	Human clinical trial (randomized/controlled) evaluating Caloric restriction and metabolic/aging outcomes.	2024

Redman LM, Belsky DW, Shalev I [43]	CALERIE™ 2 trial anal…				
Dorling JL, Belsky DW, Racette SB, Das SK, Ravussin E, Redman LM, Höchsmann C, Huffman KM, Kraus WE, Kobor MS, MacIsaac JL, Lin DTS, Corcoran DL, Martin CK [45]	Association between the FTO rs9939609 single nucleotide polymorphism and dietary adherence during a…	Caloric restriction	Review	Scholarly review on Caloric restriction, mechanisms and implications.	2021
Templeman I, Gonzalez JT, Thompson D, Betts JA [50]	The role of intermittent fasting and meal timing in weight management and metabolic health	Intermittent fasting	Review	Scholarly review on Intermittent fasting, mechanisms and implications.	2020
Stekovic, S.; Hofer, S.J.; Tripolt, N.; Aon, M.A.; Royer, P.; Pein, L.; Stadler, J.T.; Pendl, T.; Prietl, B.; Url, J.; et al [37]	Alternate day fasting improves physiological and molecular markers of aging in healthy, non-obese h…	Aging / biomarkers (general)	Review	Scholarly review on Aging / biomarkers (general), mechanisms and implications.	2020
Sun ML et al. [36]	Intermittent fasting and health	Intermittent fasting	Clinical trial (randomized/controlle d)	Human clinical trial (randomized/controlled)	2024

	outcomes: an umbrella review of systematic reviews and meta-analyse…			evaluating Intermittent fasting and metabolic/aging outcomes.	
Anton, S.D.; Lee, S.A.; Donahoo, W.T.; McLaren, C.; Manini, T.; Leeuwenburgh, C.; Pahor, M. [35]	The Effects of Time Restricted Feeding on Overweight, Older Adults: A Pilot Study	Intermittent fasting	Review	Scholarly review on Intermittent fasting, mechanisms and implications.	2019
Wilhelmi de Toledo, F.; Grundler, F.; Bergouignan, A.; Drinda, S; Michalsen, A [38]	Safety, health improvement and well-being during a 4 to 21-day fasting period in an observational s…	Aging / biomarkers (general)	Review	Scholarly review on Aging / biomarkers (general), mechanisms and implications.	2019
Madeo F, Carmona-Gutierrez D, Hofer SJ, Kroemer G [31]	Caloric restriction mimetics against age-associated disease: targets, mechanisms, and therapeutic p…	Caloric restriction	Review	Scholarly review on Caloric restriction, mechanisms and implications.	2019
Wei W, Ji S, Shang G, Zhang R [51]	Caloric restriction mimetics:	Caloric restriction	Review	Scholarly review on Caloric restriction, mechanisms and implications.	2024

	natural and synthetic compounds that mimic calorie restriction				
García-Martínez BI, Escobedo G, Chavira-Suárez E, et al. [47]	Resveratrol supplementation increases SIRT1 and antioxidant capacity in older adults: a randomized …	CR mimetics / FMD	Clinical trial (randomized/controlled)	Human clinical trial (randomized/controlled) evaluating CR mimetics / FMD and metabolic/aging outcomes.	2023
Guo M, Mi J, Jiang QM, Xu JM, Tang YY, Tian G, Wang B [46]	Metformin may produce antidepressant effects through improvement of cognitive function among depres…	CR mimetics / FMD	Review	Scholarly review on CR mimetics / FMD, mechanisms and implications.	2014
Belsky DW, Caspi A, Arseneault L, Baccarelli A, Corcoran DL, Gao X, et al. [55]	Quantification of the pace of biological aging in humans through a blood test: DunedinPACE	Aging / biomarkers (general)	Review	Scholarly review on Aging / biomarkers (general), mechanisms and implications.	2022
Kulkarni AS, Gubbi S, Barzilai N [32]	Benefits of metformin in attenuating the	CR mimetics / FMD	Review	Scholarly review on CR mimetics / FMD, mechanisms and implications.	2020

	hallmarks of aging				
Kraus D, Yang Q, Kong D, [52]	Rapamycin and gut microbiota: the emerging links	CR mimetics / FMD	Review	Scholarly review on CR mimetics / FMD, mechanisms and implications.	2023
Colman, R.J.; Anderson, R.M.; Johnson, S.C.; Kastman, E.K.; Kosmatka, K.J.; Beasley, T.M.; Allison, D.B.; Cruzen, C.; Simmons, H.A.; Kemnitz, J.W.; et al [56]	Caloric restriction delays disease onset and mortality in rhesus monkeys	Caloric restriction	Preclinical / animal study	Preclinical evidence on mechanisms relevant to Caloric restriction.	2009
Mattison JA, Roth GS, Beasley TM, Tilmont EM, Handy AM, Herbert RL, Longo DL, Allison DB, Young JE, Bryant et al [34]	Impact of caloric restriction on health and survival in rhesus monkeys from the NIA study	Caloric restriction	Preclinical / animal study	Preclinical evidence on mechanisms relevant to Caloric restriction.	2017
Levine, M.E.; Lu, A.T.; Quach, A.; Chen, B.H.; Assimes, T.L.; Bandinelli, S.; Hou, L.; Baccarelli, A.A.; Stewart, J.D.; Li, Y.; et al [33]	An epigenetic biomarker of aging for lifespan and healthspan	Aging / biomarkers (general)	Review	Scholarly review on Aging / biomarkers (general), mechanisms and implications.	2018

Belsky, D.W.; Caspi, A.; Corcoran, D.L.; Sugden, K.; Poulton, R.; Arseneault, L.; Baccarelli, A.; Chamarti, K.; Gao, X.; Hannon, E.; et al [58]	Quantification of the pace of biological aging in humans through a blood test, the DunedinPoAm DNA methylation algorithm eLife 9:e54870	Aging / biomarkers (general)	Review	Scholarly review on Aging / biomarkers (general), mechanisms and implications.	2020
Waziry R, Levine ME, Belsky DW, Newman JC, Wang J, Smith CJ, et al. [41]	Caloric restriction slows epigenetic aging in humans: a randomized controlled trial	Caloric restriction	Clinical trial (randomized/controlle d)	Human clinical trial (randomized/controlled) evaluating Caloric restriction and metabolic/aging outcomes.	2023
Lehallier, B.; Gate, D.; Schaum, N.; Nanasi, T.; Lee, S.E.; Yosef; H, Moran, P. Bernidk, D;Keller, A;Verghese, J et al [57]	Undulating changes in human plasma proteome profiles across the lifespan	Aging / biomarkers (general)	Review	Scholarly review on Aging / biomarkers (general), mechanisms and implications.	2019
Fang EF, Scheibye-Knudsen M, Jahn HJ, Li J, Ling L, Guo H, et al [61]	A research agenda for aging in China in the 21st century	Aging / biomarkers (general)	Review	Scholarly review on Aging / biomarkers (general), mechanisms and implications.	2015
Hagg S, Jylhava J [62]	Sex differences in biological aging with a	Aging / biomarkers (general)	Review	Scholarly review on Aging / biomarkers (general), mechanisms and implications.	2021

	focus on human studies				
[63] Bischoff-Ferrari HA, de Godoi Rezende Costa Molino C, Rival S, Vellas B, Rizzoli R, Kressig RW, Kanis JA, Manson JE, Dawson-Hughes B, Orav EJ, et al. [63]	The DO-HEALTH trial: design and baseline characteristics of a study of vitamin D, omega-3s, and exe…	Aging / biomarkers (general)	Clinical trial (randomized/controlled)	Human clinical trial (randomized/controlled) evaluating Aging / biomarkers (general) and metabolic/aging outcomes.	2019
Trudel-Fitzgerald C, Poole EM, Rosner B, Tworoger SS, Kubzansky LD [65]	DNA methylation-based age acceleration and risk of colorectal cancer: data from three prospective c…	Aging / biomarkers (general)	Cohort / longitudinal study	Human cohort / longitudinal study examining associations for Aging / biomarkers (general).	2020
Redman LM, Ravussin E [66]	Caloric restriction in humans: impact on physiological, psychological, and behavioral outcomes	Caloric restriction	Review	Scholarly review on Caloric restriction, mechanisms and implications.	2011
Ferrucci L, Fabbri E [67]	Inflammageing: chronic inflammation in ageing,	Aging / biomarkers (general)	Review	Scholarly review on Aging / biomarkers (general), mechanisms and implications.	2018

	cardiovascular disease, and frailty				
Most J, Tosti V, Redman LM, Fontana L [68]	Calorie restriction in humans: An update	Aging / biomarkers (general)	Review	Scholarly review on Aging / biomarkers (general), mechanisms and implications.	2017
Bohn B, Herbst A, Pfeifer M, Krakow D, Zimny S, Kopp F, Melmer A, Steinacker JM, Holl RW [73]	Impact of structured diabetes education on adherence to lifestyle changes: A longitudinal analysis	Aging / biomarkers (general)	Cohort / longitudinal study	Human cohort / longitudinal study examining associations for Aging / biomarkers (general).	2015
Wells R, Cimino JJ, Shortliffe EH [74]	Clinical decision support systems in healthcare: overview and future directions	Aging / biomarkers (general)	Review	Scholarly review on Aging / biomarkers (general), mechanisms and implications.	2020
Bailey SR, O'Malley JP, Gold R, Heintzman J, Likumahuwa S, DeVoe JE [75]	Receipt of diabetes preventive services differs by insurance status at visit	Aging / biomarkers (general)	Review	Scholarly review on Aging / biomarkers (general), mechanisms and implications.	2015
Manoogian ENC, Chow LS, Taub PR, Laferrère B, Panda S [76]	Time-restricted eating and	Intermittent fasting/	Review	Scholarly review on prevention/management of metabolic diseases.	2022

	metabolic diseases	Time-restricted			
López-Otín C, Galluzzi L, Freije JMP, Madeo F, Kroemer G [40]	Metabolic control of longevity	Aging / biomarkers (general)	Review	Scholarly review on Aging / biomarkers (general), mechanisms and implications.	2016
Ferrucci L, Gonzalez-Freire M, Fabbri E, Simonsick E, Tanaka T, Moore Z, Salimi S, Sierra F, de Cabo R. [80]	Measuring biological aging in humans: A quest	Aging / biomarkers (general)	Review	Scholarly review on Aging / biomarkers (general), mechanisms and implications.	2020
Barzilai N, Crandall JP, Kritchevsky SB, Espeland MA [55]	Metformin as a tool to target aging	CR mimetics / FMD	Review	Scholarly review on CR mimetics / FMD, mechanisms and implications.	2016

Abbreviations: CR, caloric restriction; IF, intermittent fasting; CRM, caloric restriction mimetic; FMD, fasting-mimicking diet; RCT, randomized controlled trial.
